# The association of alcohol consumption with mammographic density in a multiethnic urban population

**DOI:** 10.1186/s12885-015-1094-3

**Published:** 2015-03-14

**Authors:** Zoe Quandt, Julie D Flom, Parisa Tehranifar, Diane Reynolds, Mary Beth Terry, Jasmine A McDonald

**Affiliations:** 1 Department of Epidemiology, Columbia University Medical Center, Mailman School of Public Health, New York, NY USA; 2 Department of Internal Medicine, Stanford Hospital and Clinics, Stanford, CA USA; 3 Herbert Irving Comprehensive Cancer Center, Columbia University Medical Center, New York, NY USA; 4 School of Nursing, Long Island University, Brooklyn Campus, Brooklyn, NY USA

**Keywords:** Mammographic breast density, Alcohol consumption, Breast cancer

## Abstract

**Background:**

Alcohol consumption is associated with higher breast cancer risk. While studies suggest a modest association between alcohol intake and mammographic density, few studies have examined the association in racial/ethnic minority populations.

**Methods:**

We assessed dense breast area and total breast area from digitized film mammograms in an urban cohort of African American (42%), African Caribbean (22%), white (22%), and Hispanic Caribbean (9%) women (n = 189, ages 40-61). We examined the association between alcohol intake and mammographic density (percent density and dense area). We used linear regression to examine mean differences in mammographic density across alcohol intake categories. We considered confounding by age, body mass index (BMI), hormone contraceptive use, family history of breast cancer, menopausal status, smoking status, nativity, race/ethnicity, age at first birth, and parity.

**Results:**

Fifty percent currently consumed alcohol. Women who consumed >7 servings/week of alcohol, but not those consuming ≤7 servings/week, had higher percent density compared to nondrinkers after full adjustments (servings/week >7 β = 8.2, 95% Confidence Interval (CI) 1.8, 14.6; ≤7 β = -0.5, 95% CI -3.7, 2.8). There was a positive association between high alcohol intake and dense area after full adjustments (servings/week >7 β = 5.8, 95% CI -2.7, 14.2; ≤7 β = -0.1, 95% CI -4.4, 4.2). We did not observe race/ethnicity modification of the association between alcohol intake and percent density. In women with a BMI of <25 kg/m^2^, drinkers consuming >7 servings/week of alcohol had a 17% increase in percent density compared to nondrinkers (95% CI 5.4, 29.0) and there was no association in women with a BMI ≥ 25 kg/m^2^ (BMI ≥ 25-30 kg/m^2^ > 7 β = 5.1, 95% CI -8.5, 18.7 and BMI > 30 kg/m^2^ > 7 β = 0.5, 95% CI -6.5, 7.5) after adjusting for age and BMI (continuous).

**Conclusion:**

In a racially/ethnically diverse cohort, women who consumed >7 servings/week of alcohol, especially those with a BMI < 25 kg/m^2^, had higher percent density.

## Background

Breast density, or mammographic density, is one of the strongest intermediate markers for breast cancer - women with high mammographic densities have a 4-6 fold increase risk of developing breast cancer in comparison to those with low mammographic densities [1]. Unlike many breast cancer risk factors, mammographic density is modifiable. Tamoxifen and raloxifene use have been shown to decrease mammographic density and combined hormone replacement therapy has been shown to increase mammographic density in the range of 5-10% [2].

Alcohol intake has been consistently associated with breast cancer in the range of a 7-12% increased relative risk for every 10 grams per day of ethanol intake [3,4]. With few exceptions [5-7], the majority of studies suggest a modest positive association between alcohol intake and mammographic density [8-14], although in many studies the estimates have high imprecision [15-20]. Though the magnitude of association between alcohol intake and mammographic density vary based on method of mammographic density assessment, using a continuous measure of assessment, there is a 2-12% increase in mammographic density with daily alcohol intake [8,11,21]. The few studies that have examined alcohol intake and mammographic density in racially and ethnically diverse cohorts have not reported major racial/ethnic differences in the association [5,7,8,17]. Nevertheless, there is well established racial/ethnic variation in breast cancer incidence and mortality [22]. There are also race/ethnic differences in alcohol intake. According to the National Institute on Alcohol Abuse and Alcoholism, a greater proportion of African American and Hispanic women report abstaining from current alcohol intake (54% and 50%, respectively) compared to non-Hispanic white women (35%); however, weekly heavy drinking (≥8 drinks/week) is higher in African American (13%) and non-Hispanic white women (14%) than in Hispanic women (9%) [23]. Alcohol is a carcinogen with biologic activity that has direct and indirect effects on breast tissue [21,24,25].

Alcohol consumption is a modifiable breast cancer risk factor that may impact breast cancer risk via mammographic density. Breast cancer incidence and tumor characteristics show substantial variation by race/ethnicity, with Hispanic and African American women having lower odds of early stage breast cancer diagnosis and African American women experiencing higher incidence of invasive breast cancer in younger ages [22,26,27]. African American women also experience a higher prevalence of triple negative breast cancers, which is compelling given the literature suggests that alcohol intake is more strongly associated with hormone receptor positive breast cancer compared to hormone receptor negative breast cancers [28-31]. Studies also suggest racial/ethnic variation in mammographic density [7,32-42]. Given that alcohol consumption differ across racial/ethnic groups [23], understanding the associations between these factors and mammographic density in diverse population can provide insight into the contribution of modifiable risk factors for breast cancer in population subgroups and improve our etiologic and prevention research [23]. In a multiethnic cohort of women, we examined the association between alcohol consumption and mammographic density, as measured by percent density, dense area, and non-dense area.

## Methods

### Population

The *New York City Multiethnic Breast Cancer Project* is a collaborative study between Columbia University in Manhattan and Long Island University and Long Island College Hospital in Brooklyn (for details see [43]). In brief, we recruited 200 women between 2007 and 2008, ages 40-60 years, who completed an in-person interview and provided a signed medical release form to allow us to retrieve their mammograms [43]. We excluded data from 4 women whose mammograms were of poor quality or unavailable and 5 women who had a previous diagnosis of breast cancer. After these exclusions, 191 women remained eligible for the data analysis. We excluded two women who had incomplete alcohol data leaving a final sample size of 189 women. All participants provided written informed consent. The Internal Review Boards at Columbia University, Long Island University, and Long Island College Hospital in Brooklyn approved this study.

### Epidemiologic factors

We collected epidemiologic data through a 30-45 minute in-person interview. Specifically, we collected information on sociodemographic factors, body mass index (BMI) (calculated from self-reported weight and height recorded in patient’s chart), reproductive history (including menopausal status and hormone contraceptive use), and family and personal cancer history [43]. We categorized race/ethnicity groups based on self-reported data on race, Hispanic ethnicity, personal and parental birthplace as described previously [43]. We considered Caribbean women to be women who reported being born or having at least one parent born in a Caribbean country. We divided Caribbean women into African Caribbean (as defined by being from an English- or Creole-speaking African Caribbean country; e.g. Jamaica, Haiti) and Hispanic Caribbean (as defined by being from a Spanish-speaking Hispanic Caribbean country; e.g. Dominican Republic). We categorized non-Caribbean participants as non-Caribbean Hispanic, African American, white, and other race/ethnicities.

### Alcohol intake assessment

As part of the in-person interview, we asked women about their alcohol intake behaviors. We asked women if they had ever consumed alcoholic beverages such as coolers, beer, wine, champagne, or liquor at least once a month for six months or more. We defined women who responded “no” as never drinkers. Women who responded “yes” were defined as ever drinkers. Ever drinkers were then asked to consider the last 12 months and report if they had consumed coolers, beer, wine or champagne, or liquor at least once a month for six months. We considered women who reported that they did not drink during the past 12 months as former drinkers. We then asked women who reported consumption of any of the beverage types to detail the frequency of consumption and the number of servings (in ounces (oz)) usually consumed on the days they drank the particular beverage type. We calculated total weekly grams (g) of ethanol consumed based on the number of servings using the US Department of Agriculture guidelines for ethanol content (5 oz. of wine is 15.4 g ethanol, 12 oz. of beer is 13.9 g ethanol, 1.5 oz. of 80-proof distilled liquor is 14 g ethanol, and 12 oz. of wine cooler was 15.8 g ethanol). We also calculated the number of servings per week of alcohol. We categorized alcohol intake as a dichotomous variable (nondrinkers and current drinkers) and by using guidelines on nutrition and cancer prevention (nondrinkers, ≤7 servings/week, and >7 servings/week) [44,45]. We created independent dichotomous variables for current wine intake, current beer intake, and current liquor intake (non-current intake and current intake). We did not create a dichotomous variable for cooler intake because the sample size was too small to analyze separately (current intake n = 6).

### Mammographic density assessment

Details on mammographic density assessment have been described previously [43]. A single expert reader, blinded to other study data, assessed dense area and breast area from digitized film mammogram images (Kodak Lumisys Film Digitizer, Kodak LS85), using the Cumulus threshold software. We calculated percent density as the total dense area divided by the total breast area (both measured in number of pixels and converted to cm^2^), multiplied by 100. We calculated non-dense (fat tissue) area as the total breast area minus the total dense area. Ten percent of the films were read in duplicate resulting in a Pearson correlation of 0.99 for breast area and 0.9 for dense area for the repeated readings.

### Statistical analysis

We examined the distribution of sociodemographic factors and current alcohol intake by race/ethnicity (Table 1). For Table 1, we presented the four largest racial/ethnic groups: African American (referent group, n = 80), African Caribbean (n = 42), white (n = 41), and Hispanic Caribbean (n = 17). Hispanic non-Caribbean (n = 6) and other race/ethnicity (n = 3) were not included in Table 1 nor are they included in the regression analyses because there were too few participants in the groups to analyze separately. We performed separate linear regression analyses to examine the mean differences in mammographic density across alcohol intake categories in two models. As a secondary analysis, we also examined the association by modeling alcohol intake as a continuous variable. Model 1 was age-adjusted. Model 2 was additionally adjusted for confounders that altered the association between alcohol intake and any of the mammographic density measures by more than 10% in the age-adjusted model. We examined potential confounding by BMI, race/ethnicity, nativity (US-born, foreign-born), reproductive factors (e.g. age at first full term birth and parity, menopausal status, hormone contraceptive use), family history of breast cancer, and smoking status. Less than 3% of data on confounders was missing. We tested for additive interactions between alcohol intake (categorical) and race/ethnicity and alcohol intake (categorical) and BMI (continuous) with all confounders within the model with and without cross product terms. We further examined the association between the type of alcohol consumed and percent density. We used STATA 11.0 (College Station, TX) for analyses.

**Table 1:** **Distribution of sociodemographic factors and current alcohol intake, New York City Multiethnic Breast Cancer Project (n = 189); 2007-2008**

	Overall sample (N = 189) mean (SD)/n (percent)	African American (N = 80) mean (SD)/n (percent)	African Caribbean (N = 42) mean (SD)/n (percent)	White (N = 41) mean (SD)/n (percent)	Hispanic Caribbean (N = 17) mean (SD)/n (percent)
Age at interview (year)	49.98 (5.69)	50.15 (5.62)	49.49 (5.54)	50.06 (5.92)	51.53 (5.94)
BMI (kg/m^2^)^n/a^	29.78 (6.74)	31.43 (6.62)	30.06 (6.77)	25.53 (4.76)	32.26 (7.20)
Percent density	12.88 (11.44)	12.01 (10.78)	12.98 (13.18)	15.87 (11.25)	8.49 (9.00)
Dense area (cm^2^)	17.62 (14.57)	17.92 (14.70)	18.81 (16.86)	18.12 (12.79)	11.86 (10.45)
Non-dense area (cm^2^)	152.44 (86.58)	169.37 (90.91)	171.10 (104.43)	105.5 (40.64)	162.64 (70.54)
Current alcohol Intake					
Nondrinkers	95 (50.26)	37 (46.25)	26 (61.90)	16 (39.02)	12 (70.59)
Current drinkers	94 (49.74)	43 (53.75)	16 (38.10)	25 (60.98)	5 (29.41)
Alcohol intake in consumers					
grams/week	62.67 (151.73)	59.25 (84.08)	102.72 (338.13)	61.93 (57.41)	14.55 (12.07)
servings/week	4.29 (10.74)	4.04 (5.61)	7.26 (24.22)	4.11 (3.79)	1.00 (0.82)
≤7	80 (42.33)	35 (43.75)	15 (35.71)	20 (48.78)	5 (29.41)
>7	14 (7.41)	8 (10.00)	1 (2.38)	5 (12.20)	n/a
Current alcohol beverage type (servings/week)					
Wine	2.73 (4.41)	3.28 (6.28)	1.25 (1.36)	3.39 (3.38)	1.03 (0.16)
Beer	1.09 (1.29)	2.74 (2.88)	6.20 (12.19)	1.39 (1.60)	0.80 (0.49)
Liquor	4.21 (12.57)	2.63 (2.29)	35.22 (49.16)	1.23 (1.12)	0.66 (0.69)
Cooler	0.82 (0.81)	1.34 (0.88)	0.31 (0.23)	n/a	n/a

*Abbreviations:* BMI, Body mass index.

## Results

Our study sample included 189 women with an average age of 50 years (standard deviation (SD) 5.7) at the time of interview and an average BMI of 29.8 kg/m^2^ (SD 6.7), with 35% being postmenopausal (Table 1). About 13% of women had a first degree relative diagnosed with breast cancer. Over two-thirds of women reported having ever used hormonal birth control (68%) and having had children (71%) with the average age at birth at 23 years. Twenty-eight percent of women were former and 11% were current smokers.

Over one-third of the women were born outside the US (36%) and the racial/ethnic composition of the samples was as follow: African American (42.3%), African Caribbean (22.2%), white (21.7%), and Hispanic Caribbean (9.0%). Hispanic Caribbean women had lower percent density than Hispanic non-Caribbean women; therefore, we chose not to combine these groups (mean (SD) 8.5 (9.00) and 18.8 (12.8) (*P* = 0.04), respectively). White women had a lower average BMI (BMI = 25.5 kg/m^2^) compared to African American women (BMI = 31.4 kg/m^2^). There were no differences by race/ethnicity for percent density or dense area but white women had lower non-dense (fat) area compared to African American, African Caribbean, and Hispanic Caribbean women (*P* <0.01).

Nondrinkers included 95 women of which 69 had never consumed alcohol (36.5%) and 26 were former alcohol consumers (13.8%) (Table 1). The average (SD) alcohol intake for current drinkers was 62.7 (151.7) grams of ethanol per week or about 4.3 servings/week. Although the average (SD) percent density (13.9 (11.9)) and dense area (18.5 (15.0)) for current alcohol consumers was higher compared to non-current alcohol consumers (percent density 11.8 (10.9) and dense area 16.7 (14.1)), the comparisons were not significant (all *P values* >0.05). The majority of US born women were current drinkers (white 61% and African American 54%) in contrast to Caribbean born women (African Caribbean 38% and Hispanic Caribbean 29%). African American, African Caribbean, and white women had a higher weekly intake of alcohol compared to Hispanic Caribbean women (mean range 59.3-102.7 versus 14.6 g/week, respectively). Although less than 40% of African Caribbean women drank alcohol, those that drank had the highest weekly servings of alcohol (7.3 servings/week). The majority of alcohol consumers were wine drinkers (70%) with far fewer women who reported beer (37%), liquor (32%), or cooler (6%) intake. African Caribbean women on average consumed a greater amount of liquor (mean (SD) 35.2 (49.2) servings/week) compared to African American women (mean (SD) 2.6 (2.3) servings/week); however, this was driven by one African Caribbean woman reporting 70 servings/week of liquor. As our primary exposure construct was categorical, this value does not alter the estimates. When our construct is modeled continuously (g/week), this value does not change overall estimates.

Women who consumed >7 servings/week of alcohol, but not those drinking ≤7 servings/week, had higher mean percent density in comparison to nondrinkers after adjusting for age and BMI (servings/week >7 β = 6.9, 95% CI 1.1, 12.8; ≤7 β = -0.4, 95% CI -3.4, 2.7). The associations remained in the fully adjusted models (Table 2). Similarly, women who consumed >7 servings/week of alcohol had an 8 cm^2^ larger dense area compared to nondrinkers (servings/week >7 β = 8.3, 95% CI 0.5, 16.1; ≤7 β = 0.6, 95% CI -3.7, 4.8) in the age adjusted model; the association was attenuated after adjusting for BMI, hormone contraceptive use, family history of breast cancer, menopausal status, current smoking status, nativity, race/ethnicity, age at first birth centered at the mean, and parity (servings/week >7 β = 5.8, 95% CI -2.7, 14.2; ≤7 β = -0.1, 95% CI -4.4, 4.2). Alcohol consumption was not associated with non-dense area in fully adjusted models (servings/week >7 β = -2.3, 95% CI -43.2, 38.6; ≤7 β = 11.8, 95% CI –8.8, 32.4). In addition to including alcohol as a categorical variable (using a standard cut point reported in other papers), we modeled alcohol consumption as a continuous variable. We observed a linear positive relationship between alcohol intake (g/week) and percent density after fully adjusting for confounders (β = 0.03, 95% CI 0.002, 0.06), but found no association between alcohol intake and dense area (β = 0.03, 95% CI -0.01, 0.07) or non-dense area (β = -0.03, 95% CI -0.2, 0.2).

**Table 2:** **Multiple linear regression for mammographic density and current alcohol intake, New York City Multiethnic Breast Cancer Project (n = 180); 2007-2008**

	Model 1^a^			Model 2^b^		
	β	95% CI	*P*	β	95% CI	*P*
*Percent density*						
Current alcohol intake						
Nondrinkers	0	reference		0	reference	
≤7 servings/week	0.54	−2.75, 3.84	0.75	−0.46	−3.69, 2.78	0.78
>7 servings/week	8.95	2.89, 15.00	<0.01	8.22	1.81, 14.64	0.01
*Dense area*						
Current alcohol intake						
Nondrinkers	0	reference		0	reference	
≤7 servings/week	0.56	−3.68, 4.79	0.80	−0.10	−4.38, 4.18	0.96
>7 servings/week	8.30	0.52, 16.08	0.04	5.75	−2.73, 14.24	0.18
*Non-dense area*						
Current alcohol intake						
Nondrinkers	0	reference		0	reference	
≤7 servings/week	−0.44	−27.15, 26.28	0.97	11.82	−8.81, 32.44	0.26
>7 servings/week	−19.65	−68.77, 29.46	0.43	−2.33	−43.21, 38.56	0.91

*Abbreviations:* CI, Confidence interval.

^a^Model 1 is adjusted for age at interview (years).

^b^Model 2 is adjusted for age at interview, BMI (continuous, kg/m^2^), hormone contraceptive use (ever use vs never use), family history of breast cancer (yes or no), menopausal status (pre- or post-), current smoking status, nativity (US or foreign-born), race/ethnicity, age at first birth centered at the mean, and parity.

**Figure 1:**
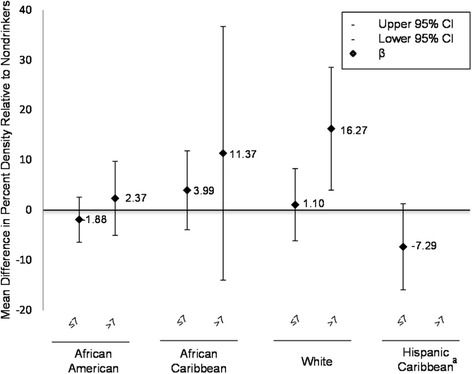
**Multiple linear regression coefficients for the association between percent density and current alcohol intake (servings/week) by race/ethnicity, New York City Multiethnic Breast Cancer Project (n=176); 2007-2008.** Models are adjusted for age at interview (years) and BMI (kg/m^2^, continuous). ^a^ Hispanic Caribbean women do not report consuming >7 servings/week of alcohol.

In race/ethnic-stratified analyses after adjusting for age and continuous BMI, the confounders specific to percent density, we observed no associations between alcohol intake and percent density in African American, African Caribbean, and Hispanic women (Figure 1). White women who consumed >7 servings/week of alcohol had a 16% increase in percent density (95% CI 4.0, 28.5) after adjusting for age and BMI; however, only 5 women reported consuming at this level. Results for race/ethnic stratified analyses were essentially the same after fully adjusting for confounders where the strongest association was observed in white women who consumed >7 servings/week of alcohol (β = 29.9, 95% CI 18.2, 41.6). We also observed a strong positive linear relationship between alcohol intake (g/week) and percent density in fully adjusted models in white women only (β = 0.09, 95% CI 0.004, 0.2). There was no additive interaction between alcohol intake and race/ethnicity when examining percent density, dense area, or non-dense area (all *P values* >0.05). However, race/ethnic stratified analyses for dense area suggest stronger effects in white women and African Caribbean women (data not shown).

**Figure 2:**
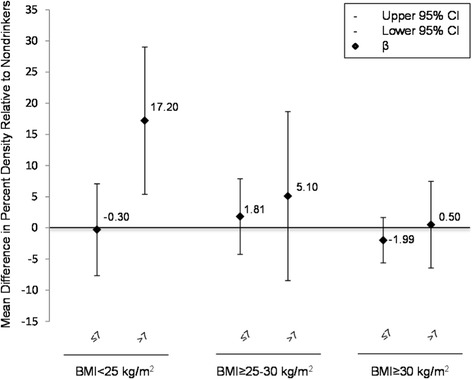
**Multiple linear regression coefficients for the association between percent density and current alcohol intake (servings/week) by BMI, New York City Multiethnic Breast Cancer Project (n=176); 2007-2008.** Models are adjusted for age at interview (years) and BMI (kg/m^2^, continuous).

In BMI-stratified analyses adjusted for age and continuous BMI, percent density specific confounders, in women with a BMI of <25 kg/m^2^, those who consumed >7 servings/week of alcohol had a 17% increase in percent density (95% CI 5.4, 29.0) (Figure 2). There was no association between percent density and alcohol consumption in women with a BMI = 25- < 30 kg/m^2^ (servings/week ≤7 β = 1.8, 95% CI -4.3, 7.9; >7 β = 5.1, 95% CI -8.5, 18.7) or BMI ≥ 30 kg/m^2^ (servings/week ≤7 β = -2.0, 95% CI -5.6, 1.7; >7 β = 0.5, 95% CI -6.5, 7.5). Results for BMI-stratified analyses were essentially the same after fully adjusting for all confounders (data not shown). Further, results were confirmed in fully adjusted models where we also observed a positive linear relationship between alcohol intake modeled as a continuous variable (g/week) and percent density in women with a BMI of <25 kg/m^2^ (β = 0.07, 95% CI 0.002, 0.1) and null associations in women with a BMI ≥ 25 kg/m^2^ (BMI = 25- < 30 kg/m^2^ β = -0.1, 95% CI -0.1, 0.08 and BMI ≥ 30 kg/m^2^ β = 0.02, 95% CI -0.01, 0.05). We observed statistical interaction between alcohol intake and BMI on an additive scale when we modeled for percent density (*P* <0.01). In contrast, we observed no interaction with BMI when we examined dense area or non-dense area. However, similar to percent density after adjusting for age and BMI, when we modeled for dense area we observed stronger associations in women with a BMI of <25 kg/m^2^. Women consuming >7 servings/week of alcohol had greater dense area, but the association did not reach significance (*P* = 0.07).

We examined the association between alcohol beverage type and percent density because we observed an association between alcohol intake and percent density in our main analyses. In comparison to women who did not consume wine, wine intake was associated with a 4.1% increase in percent density in the age-adjusted model (95% CI 0.7, 7.4); however, the association was attenuated after adjusting for BMI, beer intake, liquor intake, and cooler intake (β = 1.9, 95% CI -1.4, 5.3). We observed no association between beer intake and percent density (β = 0.7, 95% CI -3.3, 4.7) or liquor intake and percent density (β = -0.3, 95% CI -4.4, 3.8) when adjusted for age, BMI, and intake of other types of alcohol.

## Discussion

We observed that high levels of alcohol consumption (>7 servings/week) were associated with an increase in percent density, and though results for dense area were similar, but not statistically significant, an increase in dense area. We found no association between alcohol intake and non-dense area. Our results for linear associations between mammographic density measures and alcohol intake in g/week confirmed these findings. Although we were limited with our overall sample size and cannot conclude modification by race/ethnicity, the race/ethnic-stratified analyses did suggest stronger associations between alcohol intake and percent density in white women. We also found women with a BMI < 25 kg/m^2^ may be at greater risk for higher mammographic density associated with alcohol intake.

Our study observed an approximate 8% increase in the relative amount of mammographic density with high alcohol intake, which is within range of other breast cancer risk factors known to modify mammographic density (range 2-10%) [1,2]. With few exceptions [5,46,47], our findings are consistent with prior studies that show a positive association between mammographic density and current alcohol intake [8,9,11,17,18,48-50], although the precision for many of these estimates is highly variable [21]. In studies that examine beverage type [8,51-53], the majority confirm a positive association between wine intake and mammographic density [8,9,18]. We found a positive association between wine intake and percent density; however, the association was attenuated after adjusting for age and BMI. The inconsistencies across studies may be attributed to a variety of factors, including but not limited to, different methods of measuring mammographic density (i.e. BI-RADS and Wolfe classification versus the continuous measures as also used in our study), low levels of alcohol intake, alcohol’s varied roles in carcinogenesis, and additional factors that modify alcohol’s effect (as reviewed in [21]). First, studies using BI-RADS and Wolfe classification patterns capture variation in the extent of mammographic density across only four categories; therefore, studies using these categorical measures of mammographic density require larger changes to reflect a categorical change. In contrast, studies capturing variation in mammographic density through continuous measures that are based on computer threshold programs require smaller changes to reflect mammographic density change. Second, some of the strongest findings for the association between mammographic density and alcohol intake have been in populations with high levels of alcohol consumption. While the Norwegian cohort with an average consumption of 6 g/day alcohol did not find an association with percent density [46], in a Mediterranean population with consumption levels at the highest tertile starting at 12.0 g/day of alcohol, a positive association was observed in pre- and post-menopausal women [9]. Similarly, in the New York site of the National Collaborative Perinatal Project (NCPP) cohort with a reported average consumption of 4.37 servings/week (estimated consumption of 6.2 g/day), alcohol intake was positively associated with mammographic density [8]. Third, alcohol is known to play multiple mechanistic roles in carcinogenesis that are not measured in our analysis [21], such as the evidence for the role of insulin-like growth factor-1 (IGF-1). Studies suggest that alcohol can increase IGF-1 levels by stimulation of the liver post-alcohol consumption [54]. Furthermore, increased IGF-1 has been associated with increased breast cancer risk [55-58], mammographic density (especially in premenopausal women) [59-61], and alcohol consumption [62]. Alcohol can also increase circulating estrogen levels and there is a positive association between circulating estrogen and breast cancer development. Estrogen may induce hormone mediated cell proliferation that can result in genetic alterations and greater opportunity for genetic damage [21,63]. The variability in studies examining the association between mammographic density and current alcohol could be due to IGF-1 or circulating estrogen levels. Future epidemiological studies with biomarker availability are needed to explore this further. Lastly, the inconsistencies across studies may be attributed to study population variation in factors such as genetic make-up (i.e. alcohol metabolizing genes), diet (i.e. folate), and sociodemographic and hormonal factors (i.e. BMI, menopausal status, HRT) that have all been associated with modifying alcohol’s effect on breast cancer risk [21,54].

Studies are inconsistent as to whether mammographic density varies across race/ethnicity [7,32-42]. While none of our cross-product terms between alcohol intake and race/ethnicity reached statistical significance, we observed stronger associations between current alcohol intake and mammographic density in whites. However, we are limited in the ability to test for interactions by race/ethnicity due to small sample sizes as very limited numbers of women consumed more than 7 servings/week of alcohol. Nevertheless, similar to other studies [5,8], we found no association between mammographic density and alcohol intake among Hispanic women. In the Chicago Breast Health Project that consists of 296 Hispanic women, alcohol intake was not associated with mammographic density [5]. The lack of an association may have been attributed to very low levels of alcohol intake given that only 22% of women reported consuming more than 1 serving/week. Hispanic Caribbean women in our cohort also reported low levels of alcohol consumption with an average intake of 1 serving/week. The association between alcohol intake and mammographic density has been rarely studied among women of African descent but in the NCPP cohort, a positive association was reported for increasing current alcohol intake and increasing mammographic density in African American women [8]. In our study, despite our African American women having similar alcohol intake levels as white women, there was no association between alcohol intake and percent density in African American women. Breast cancer mortality is higher among African American women [64], and women who immigrate to the United States and their female offspring also experience an increased risk of breast cancer [65]. Large epidemiological studies of a heterogeneous population of women of African descent are needed to understand the association between alcohol intake and mammographic density, two modifiable breast cancer risk factors.

Limited studies ascertain the association between alcohol and multiple measures of mammographic density [21]. While our dense area results also suggested a positive relationship, the association did not reach statistical significance. Percent density is a proportion of the total breast area and is greatly influenced by body size; whereas, dense area is less influenced by body size. Our cohort had a higher average BMI (Mean (SD) 29.8 (6.7)) than the NCPP cohort (mean (SD) 27.6 (6.5)), and these differences in BMI may account for the different results across the two studies suggesting that body size may impact our overall findings. We observed a stronger positive association between alcohol intake and percent density in women with a BMI < 25 kg/m^2^ than in women with higher BMI. This is consistent with multiple breast cancer studies that show the positive association between lifetime alcohol intake and breast cancer risk is strongest in leaner women [66-68]. In a cohort of postmenopausal Norwegian women, there was no association between alcohol intake and mammographic density across tertiles of BMI [46]. In a multiethnic cohort of white, African American, and Asian American women, the positive associations between mammographic density and breast cancer risk were stronger in the leanest women and the most obese women [41]. If associations are strongest in the leanest women, given that all of our women with a BMI > 25 kg/m^2^ were women of African descent, this may explain why we do not see a strong association between high levels of alcohol intake (>7 servings/week) and mammographic density in women of African descent. Fat tissue can contribute to estrogen production which can lead to increased breast cancer risk [69-71]. Therefore, future studies should stratify or select based on BMI to further better understand the contribution of alcohol consumption to mammographic density across BMI level.

Limitations of our study include the possibility of information bias due to self-reported alcohol intake. However, women being screened likely did not know their mammographic density resulting in non-differential bias. People are also known to under-report alcohol intake, which would result in an under-estimation of the magnitude of association. Our study is limited in the number of women who reported consuming >7 servings/week (n = 14), which contributed to large confidence intervals. However, when we modeled alcohol intake as a continuous variable we confirmed the positive relationship between alcohol intake and mammographic density. We also acknowledge that given the estimates observed for white women are similar to the estimates observed for women with a BMI < 25 kg/m^2^; these two analyses may be capturing similarities and in fact for women consuming >7 servings/week of alcohol with a BMI < 25 kg/m^2^, 4 of the 5 women were white. Further, we are unable to examine the associations between alcohol intake and mammographic density stratified by nativity given the small number of women that consume high amounts of alcohol (>7 servings/week) and are born outside the US (n = 2). We also did not assess levels of alcohol intake in earlier life periods; however, many studies have shown that current alcohol, and not past alcohol intake, is associated with increased mammographic density [8,19,50].

## Conclusions

Mammographic density is one of the strongest intermediate markers for breast cancer risk and is regularly clinically screened at mammography visits. With over one-third of states passing a version of the Breast Density Notification Law that mandates release of high mammographic density information to women, women may begin to seek information on how to modify their mammographic density to reduce their breast cancer risk. Alcohol consumption has been consistently associated with breast cancer risk and our study supports an association with increased mammographic density. Future studies should evaluate whether decreasing alcohol intake is associated with a reduction in mammographic density. Identifying women at higher risk of breast cancer because of their mammographic density would be an important time to reinforce prevention messages about alcohol intake. Further, investigating differences in alcohol and mammographic density association in women with different body size and racial and ethnic backgrounds can inform etiologic research as well as prevention efforts.

## References

[1] Singletary K, Nelshoppen J, Wallig M (1995). Enhancement by chronic ethanol intake of N-methyl-N-nitrosourea-induced rat mammary tumorigenesis. Carcinogenesis.

[2] Martin LJ, Boyd NF (2008). Mammographic density. Potential mechanisms of breast cancer risk associated with mammographic density: hypotheses based on epidemiological evidence. Breast Cancer Res.

[3] Prevention. CfDCa (2011). Prevalence of Coronary Heart Disease - United States, 2006-2010. Morb Mortal Wkly Rep.

[4] Allen NE, Beral V, Casabonne D, Kan SW, Reeves GK, Brown A (2009). Moderate alcohol intake and cancer incidence in women. J Natl Cancer Inst.

[5] Gapstur SM, Lopez P, Colangelo LA, Wolfman J, Van Horn L, Hendrick RE (2003). Associations of breast cancer risk factors with breast density in Hispanic women. Cancer Epidemiol Biomarkers Prev.

[6] Brisson J, Verreault R, Morrison AS, Tennina S, Meyer F (1989). Diet, mammographic features of breast tissue, and breast cancer risk. Am J Epidemiol.

[7] Maskarinec G, Pagano I, Chen Z, Nagata C, Gram IT (2007). Ethnic and geographic differences in mammographic density and their association with breast cancer incidence. Breast Cancer Res Treat.

[8] Flom JD, Ferris JS, Tehranifar P, Terry MB (2009). Alcohol intake over the life course and mammographic density. Breast Cancer Res Treat.

[9] Masala G, Ambrogetti D, Assedi M, Giorgi D, Del Turco MR, Palli D (2006). Dietary and lifestyle determinants of mammographic breast density. A longitudinal study in a Mediterranean population. Int J Cancer.

[10] Vachon CM, Kuni CC, Anderson K, Anderson VE, Sellers TA (2000). Association of mammographically defined percent breast density with epidemiologic risk factors for breast cancer (United States). Cancer Causes Control.

[11] Boyd NF, Connelly P, Byng J, Yaffe M, Draper H, Little L (1995). Plasma lipids, lipoproteins, and mammographic densities. Cancer Epidemiol Biomarkers Prev.

[12] Boyd NF, McGuire V, Fishell E, Kuriov V, Lockwood G, Tritchler D (1989). Plasma lipids in premenopausal women with mammographic dysplasia. Br J Cancer.

[13] Stevens VL, McCullough ML, Pavluck AL, Talbot JT, Feigelson HS, Thun MJ (2007). Association of polymorphisms in one-carbon metabolism genes and postmenopausal breast cancer incidence. Cancer Epidemiol Biomarkers Prev.

[14] Knight JA, Vachon CM, Vierkant RA, Vieth R, Cerhan JR, Sellers TA (2006). No association between 25-hydroxyvitamin D and mammographic density. Cancer Epidemiol Biomarkers Prev.

[15] Pankow JS, Vachon CM, Kuni CC, King RA, Arnett DK, Grabrick DM (1997). Genetic analysis of mammographic breast density in adult women: evidence of a gene effect. J Natl Cancer Inst.

[16] Rosamond WD, Chambless LE, Heiss G, Mosley TH, Coresh J, Whitsel E (2012). Twenty-two-year trends in incidence of myocardial infarction, coronary heart disease mortality, and case fatality in 4 US communities, 1987-2008. Circulation.

[17] Maskarinec G, Takata Y, Pagano I, Lurie G, Wilkens LR, Kolonel LN (2006). Alcohol consumption and mammographic density in a multiethnic population. Int J Cancer.

[18] Vachon CM, Kushi LH, Cerhan JR, Kuni CC, Sellers TA (2000). Association of diet and mammographic breast density in the Minnesota breast cancer family cohort. Cancer Epidemiol Biomarkers Prev.

[19] Vachon CM, Sellers TA, Janney CA, Brandt KR, Carlson EE, Pankratz VS (2005). Alcohol intake in adolescence and mammographic density. Int J Cancer.

[20] Funkhouser E, Waterbor JW, Cole P, Rubin E (1993). Mammographic patterns and breast cancer risk factors among women having elective screening. South Med J.

[21] McDonald J, Goyal A, Terry M (2013). Alcohol Intake and Breast Cancer Risk: Weighing the Overall Evidence. Curr Breast Cancer Rep.

[22] Chlebowski RT, Chen Z, Anderson GL, Rohan T, Aragaki A, Lane D (2005). Ethnicity and Breast Cancer: Factors Influencing Differences in Incidence and Outcome. J Natl Cancer Inst.

[23] Qi X, Ma X, Yang X, Fan L, Zhang Y, Zhang F (2010). Methylenetetrahydrofolate reductase polymorphisms and breast cancer risk: a meta-analysis from 41 studies with 16,480 cases and 22,388 controls. Breast Cancer Res Treat.

[24] Dumitrescu RG, Shields PG (2005). The etiology of alcohol-induced breast cancer. Alcohol.

[25] Singletary KW, Gapstur SM (2001). Alcohol and breast cancer: review of epidemiologic and experimental evidence and potential mechanisms. JAMA.

[26] Johnson RH, Chien FL, Bleyer A (2013). Incidence of breast cancer with distant involvement among women in the United States, 1976 to 2009. JAMA.

[27] Iqbal J, Ginsburg O, Rochon PA, Sun P, Narod SA (2015). Differences in breast cancer stage at diagnosis and cancer-specific survival by race and ethnicity in the United States. JAMA.

[28] Fagherazzi G, Vilier A, Boutron-Ruault MC, Mesrine S, Clavel-Chapelon F: Alcohol consumption and breast cancer risk subtypes in the E3N-EPIC cohort. Eur J Cancer Prev. 2014.Publshed online ahead of print. 10.1097/CEJ.0000000000000031 24743350

[29] Li CI, Chlebowski RT, Freiberg M, Johnson KC, Kuller L, Lane D (2010). Alcohol consumption and risk of postmenopausal breast cancer by subtype: the women’s health initiative observational study. J Natl Cancer Inst.

[30] Boyle P (2012). Triple-negative breast cancer: epidemiological considerations and recommendations. Ann Oncol.

[31] Kabat GC, Kim M, Phipps AI, Li CI, Messina CR, Wactawski-Wende J (2011). Smoking and alcohol consumption in relation to risk of triple-negative breast cancer in a cohort of postmenopausal women. Cancer Causes Control.

[32] del Carmen MG, Halpern EF, Kopans DB, Moy B, Moore RH, Goss PE (2007). Mammographic breast density and race. AJR Am J Roentgenol.

[33] Zhang J, Qiu LX, Wang ZH, Wu XH, Liu XJ, Wang BY (2010). MTHFR C677T polymorphism associated with breast cancer susceptibility: a meta-analysis involving 15,260 cases and 20,411 controls. Breast Cancer Res Treat.

[34] Vachon CM, van Gils CH, Sellers TA, Ghosh K, Pruthi S, Brandt KR (2007). Mammographic density, breast cancer risk and risk prediction. Breast Cancer Res.

[35] Lissowska J, Gaudet MM, Brinton LA, Chanock SJ, Peplonska B, Welch R (2007). Genetic polymorphisms in the one-carbon metabolism pathway and breast cancer risk: a population-based case-control study and meta-analyses. Int J Cancer.

[36] Yu L, Chen J (2012). Association of MHTFR Ala222Val (rs1801133) polymorphism and breast cancer susceptibility: An update meta-analysis based on 51 research studies. Diagn Pathol.

[37] Izmirli M (2013). A literature review of MTHFR (C677T and A1298C polymorphisms) and cancer risk. Mol Biol Rep.

[38] Halsted CH, Villanueva JA, Devlin AM, Chandler CJ (2002). Metabolic interactions of alcohol and folate. J Nutr.

[39] Maskarinec G, Pagano I, Lurie G, Kolonel LN (2006). A longitudinal investigation of mammographic density: the multiethnic cohort. Cancer Epidemiol Biomarkers Prev.

[40] McCormack VA, Perry N, Vinnicombe SJ, Silva IS (2008). Ethnic Variations in Mammographic Density: A British Multiethnic Longitudinal Study. Am J Epidemiol.

[41] De Vogli R, Chandola T, Marmot MG (2007). Negative aspects of close relationships and heart disease. Arch Intern Med.

[42] Razzaghi H, Troester M, Gierach G, Olshan A, Yankaskas B, Millikan R (2012). Mammographic density and breast cancer risk in White and African American Women. Breast Cancer Res Treat.

[43] Tehranifar P, Reynolds D, Flom J, Fulton L, Liao Y, Kudadjie-Gyamfi E (2011). Reproductive and menstrual factors and mammographic density in African American, Caribbean, and white women. Cancer Causes Control.

[44] Mao Q, Gao L, Wang H, Wang Q, Zhang T. The Alcohol Dehydrogenase 1C(rs698) Genotype and Breast Cancer: A Meta-analysis. Asia Pac J Public Health. Published online before print. May 31, 2012 10.1177/1010539512446962 22652248

[45] World Cancer Research Fund/American Institute of Cancer Research (2007). Food, nutrition, physical activity, and the prevention of cancer: a global perspective.

[46] Qureshi SA, Couto E, Hofvind S, Wu AH, Ursin G (2012). Alcohol intake and mammographic density in postmenopausal Norwegian women. Breast Cancer Res Treat.

[47] Tseng M, Byrne C, Evers KA, Daly MB (2007). Dietary intake and breast density in high-risk women: a cross-sectional study. Breast Cancer Res.

[48] Yaghjyan L, Mahoney MC, Succop P, Wones R, Buckholz J, Pinney SM (2012). Relationship between breast cancer risk factors and mammographic breast density in the Fernald Community Cohort. Br J Cancer.

[49] Jeon JH, Kang JH, Kim Y, Lee HY, Choi KS, Jun JK (2011). Reproductive and Hormonal Factors Associated with Fatty or Dense Breast Patterns among Korean Women. Cancer Res Treat.

[50] Cabanes A, Pastor-Barriuso R, Garcia-Lopez M, Pedraz-Pingarron C, Sanchez-Contador C, Vazquez Carrete JA (2011). Alcohol, tobacco, and mammographic density: a population-based study. Breast Cancer Res Treat.

[51] Chronic Diseases and Health Promotion. [http://www.cdc.gov/chronicdisease/overview/index.htm]

[52] Schatzkin A, Abnet CC, Cross AJ, Gunter M, Pfeiffer R, Gail M (2009). Mendelian randomization: how it can–and cannot–help confirm causal relations between nutrition and cancer. Cancer Prev Res.

[53] McCarty CA, Reding DJ, Commins J, Williams C, Yeager M, Burmester JK (2012). Alcohol, genetics and risk of breast cancer in the Prostate, Lung, Colorectal and Ovarian (PLCO) Cancer Screening Trial. Breast Cancer Res Treat.

[54] Seitz HK, Pelucchi C, Bagnardi V, La Vecchia C (2012). Epidemiology and pathophysiology of alcohol and breast cancer: Update 2012. Alcohol Alcohol.

[55] Endogenous H, Breast Cancer Collaborative G, Key TJ, Appleby PN, Reeves GK, Roddam AW (2010). Insulin-like growth factor 1 (IGF1), IGF binding protein 3 (IGFBP3), and breast cancer risk: pooled individual data analysis of 17 prospective studies. Lancet Oncol.

[56] Rinaldi S, Peeters PH, Berrino F, Dossus L, Biessy C, Olsen A (2006). IGF-I, IGFBP-3 and breast cancer risk in women: The European Prospective Investigation into Cancer and Nutrition (EPIC). Endocr Relat Cancer.

[57] Boyd NF, Stone J, Martin LJ, Jong R, Fishell E, Yaffe M (2002). The association of breast mitogens with mammographic densities. Br J Cancer.

[58] Lann D, LeRoith D (2008). The role of endocrine insulin-like growth factor-I and insulin in breast cancer. J Mammary Gland Biol Neoplasia.

[59] Boyd NF, Martin LJ, Yaffe MJ, Minkin S (2006). Mammographic density: a hormonally responsive risk factor for breast cancer. J Br Menopause Soc.

[60] Walker K, Fletcher O, Johnson N, Coupland B, McCormack VA, Folkerd E (2009). Premenopausal mammographic density in relation to cyclic variations in endogenous sex hormone levels, prolactin, and insulin-like growth factors. Cancer Res.

[61] Becker S, Kaaks R (2009). Exogenous and endogenous hormones, mammographic density and breast cancer risk: can mammographic density be considered an intermediate marker of risk?. Recent Results Cancer Res.

[62] Yu H (1999). Comment on association between insulin-like growth factor-I (IGF-I) and bone mineral density: further evidence linking IGF-I to breast cancer risk. J Clin Endocrinol Metab.

[63] Yager JD, Davidson NE (2006). Estrogen carcinogenesis in breast cancer. N Engl J Med.

[64] Hunt BR, Whitman S, Hurlbert MS (2014). Increasing Black:White disparities in breast cancer mortality in the 50 largest cities in the United States. Cancer Epidemiol.

[65] Andreeva VA, Unger JB, Pentz MA (2007). Breast cancer among immigrants: a systematic review and new research directions. J Immigr Minor Health.

[66] Terry MB, Zhang FF, Kabat G, Britton JA, Teitelbaum SL, Neugut AI (2006). Lifetime alcohol intake and breast cancer risk. Ann Epidemiol.

[67] Gapstur SM, Potter JD, Sellers TA, Folsom AR (1992). Increased risk of breast cancer with alcohol consumption in postmenopausal women. Am J Epidemiol.

[68] Schatzkin A, Jones DY, Hoover RN, Taylor PR, Brinton LA, Ziegler RG (1987). Alcohol consumption and breast cancer in the epidemiologic follow-up study of the first National Health and Nutrition Examination Survey. N Engl J Med.

[69] Morris PG, Hudis CA, Giri D, Morrow M, Falcone DJ, Zhou XK (2011). Inflammation and increased aromatase expression occur in the breast tissue of obese women with breast cancer. Cancer Prev Res.

[70] Falk RT, Gentzschein E, Stanczyk FZ, Garcia-Closas M, Figueroa JD, Ioffe OB (2012). Sex steroid hormone levels in breast adipose tissue and serum in postmenopausal women. Breast Cancer Res Treat.

[71] O’Neill JS, Elton RA, Miller WR (1988). Aromatase activity in adipose tissue from breast quadrants: a link with tumour site. Br Med J.

